# View of Pakistani Residents toward Coronavirus Disease (COVID-19) during a Rapid Outbreak: A Rapid Online Survey

**DOI:** 10.3390/ijerph17103347

**Published:** 2020-05-12

**Authors:** Khezar Hayat, Meagen Rosenthal, Sen Xu, Muhammad Arshed, Pengchao Li, Panpan Zhai, Gebrehaweria Kassa Desalegn, Yu Fang

**Affiliations:** 1 Department of Pharmacy Administration and Clinical Pharmacy, School of Pharmacy, Xi’an Jiaotong University, Xi’an 710061, China; khezar.hayat@uvas.edu.pk (K.H.); marsxs@stu.xjtu.edu.cn (S.X.); lipengchao1996@stu.xjtu.edu.cn (P.L.); emmazhai@stu.xjtu.edu.cn (P.Z.); desalegnkassa188@gmail.com (G.K.D.); 2 Center for Drug Safety and Policy Research, Xi’an Jiaotong University, Xi’an 710061, China; 3 Shaanxi Centre for Health Reform and Development Research, Xi’an 710061, China; 4 Institute of Pharmaceutical Sciences, University of Veterinary and Animal Sciences, Lahore 54000, Pakistan; 5 Department of Pharmacy Administration, School of Pharmacy, University of Mississippi, Oxford, MS 38677, USA; mmrosent@olemiss.edu; 6 Department of Community Health, Faculty of Medicine & Health Science, University of Putra Malaysia (UPM), Selangor 43400, Malaysia; drarshedchaudhary@gmail.com

**Keywords:** coronavirus, COVID-19, knowledge, infection, Pakistan

## Abstract

Background: Coronavirus disease (COVID-19) is a deadly disease that is affecting most of the countries worldwide. Public understanding, including knowledge about signs and symptoms, mode of transmission, and hygiene of COVID-19, is vital for designing effective control strategies during a public health crisis. The current study is aimed at investigating the public’s perspective about COVID-19, including their knowledge, attitude, and practices. Methods: A rapid online survey comprising 22 items was administered during the rapid outbreak of COVID-19 in Pakistan. Questions were focused on the prevention, transmission, clinical features, and control of COVID-19. In addition, the attitudes and practices of the participants were explored. Descriptive statistics, Mann–Whitney tests, Kruskal–Wallis tests, and regression analysis were carried out during data analysis. Results: A total of 1257 respondents participated in this study. Most of the respondents had good knowledge (good = 64.8%, average = 30.5%, poor = 4.7%) of COVID-19. Gender, marital status, education, and residence were observed to have a significant association with the knowledge score. A vast majority of the survey respondents (77.0%) believed that COVID-19 would be controlled successfully in Pakistan. The practices of wearing a mask (85.8%) and handwashing (88.1%) were common among the participants. Conclusion: The participants demonstrated good knowledge and reasonable attitudes and practices toward most aspects of the COVID-19 outbreak. Improvements in certain areas could be made by mass-level education.

## 1. Introduction

Coronavirus disease (officially abbreviated as COVID-19), which mainly targets the respiratory system of the body, was first detected in 2019 in Wuhan, China [1,2,3]. More than 1000 deaths have been reported in fifteen countries, including the United States, Spain, Italy, Germany, China, Iran, the UK, Belgium, the Netherlands, Canada, Sweden, Turkey, France, Brazil, and Switzerland—all attributed to COVID-19 [4]. This viral outbreak across the world has paralyzed the healthcare system of nearly every country, potentiating the risk of mortality and morbidity day by day [2]. Due to the spread of COVID-19 across the majority of countries outside China, a global pandemic was declared by the World Health Organization (WHO) on 12 March 2020 [4,5]. As of April 22, 2,594,835 confirmed cases of COVID-19, and 181,170 deaths were reported worldwide [6]. The intensity of the catastrophic effects of COVID-19 is equally faced by developing and developed nations; however, the situation could be worse in countries with fragile healthcare systems [7,8]. 

Pakistan is a low- and middle-income country with a population of 197 million. It has four provinces, namely Punjab, Sindh, Khyber Pakhtunkhwa (KPK), and Balochistan, and three territories, including Islamabad Capital Territory, Gilgit–Baltistan, Azad Jammu, and Kashmir [9]. The first two confirmed cases of COVID-19 in Pakistan were reported on 26 February 2020, which rang the bell for the upcoming storm [10]. At the time of writing this article, there were 10,076 confirmed COVID-19 cases and 212 deaths across the country (Table 1) [11]. Punjab province was severely hit by COVID-19 as it topped the ranks in terms of the number of COVID-19 cases (with 4328 cases as of 22 April 2020) [11]. The government has opted for some unprecedented strategies, including partial lockdown, social distancing, travel restrictions, suspension of public transport, setup of quarantine centers, diagnostic laboratories, and isolation wards. Despite these measures, the numbers are continuously amplifying every minute. 

The impact of COVID-19 will strongly depend on the behavior of people, which in turn will rely on their understanding of COVID-19. There is a massive spread of disinformation and misinformation about COVID-19 on various social media platforms, making it difficult for the public to determine which ones they should trust. To prevent people’s misunderstanding about this viral disease, the WHO had to launch a page entitled “myth busters” on their webpage [12]. 

The effectiveness of government-run information campaigns significantly depends on what people perceive and know about COVID-19. Therefore, it will be of great importance to educate the public about hygiene principles, the spread of diseases, and possible options to treat it. This will further help in tailoring and installing effective control measures. 

The commitment of Pakistanis to these control measures is necessary to win the battle against COVID-19, which primarily depends on their knowledge, attitude, and practices (KAP), as highlighted by the KAP theory [13,14]. Substantial efforts to contain this virus have already been made by the health authorities of Pakistan; however, education and public awareness are part and parcel among these measures as described during the spread of Severe Acute Respiratory Distress Syndrome (SARS) [15]. Therefore, the present study was designed with the goal of exploring the understanding of the public toward COVID-19. 

## 2. Methods

### 2.1. Study Design

This was an online study conducted from 29 March 2020 to 15 April 2020, after the partial lockdown of Pakistan. We opted for an online platform to collect the data since it was difficult to conduct a community-based survey during this time. In Pakistan, 76 million people have internet access, and 37 million people are actively using various social media platforms [16]. 

### 2.2. Survey Instrument

A thorough literature survey of relevant articles and guidelines was conducted to design the survey instrument [17,18,19,20,21]. Once the survey conceptualization was completed, the face and content validity of the instrument was tested by an expert team comprising two professors with a background in pharmacy practice. Small changes in the wording were made, as suggested by the experts, to enhance understanding of the items by the participants. 

The approved version of the instrument had 22 items and comprised four parts. The first part contained questions related to the demographic information of the participants, such as gender, age, marital status, education, occupation, and current residence. To investigate the knowledge of the participants, 11 questions were asked in the second part with three options: “yes”, “no”, and “do not know”. These knowledge-based questions were based on signs and symptoms, modes of transmission, and strategies to prevent the transmission of COVID-19. The third part had two questions focused on the attitude toward COVID-19. For the first question, “Do you agree that COVID-19 will finally be successfully controlled?”, a 5-point Likert scale (strongly agree, agree, neutral, disagree, and strongly disagree) was used. Whereas, the second question, “Do you have confidence that Pakistan can win the battle against the COVID-19 virus?”, was asked with three options, including “yes”, “no”, and “maybe”. The last part contained the options “yes” and “no” for three questions about the practices of the participants toward COVID-19, such as their recent visit to the crowded area, regular hand washing, and the wearing of face masks. The reliability of the instrument was checked by determining the value of Cronbach’s α, which was more than 7, indicating an acceptable level of internal consistency [22].

The knowledge score of the participants was calculated by assigning one point to each correct item. The range of overall knowledge was 0 to 11. The participants were considered to have poor, average, or good knowledge if their score was <5, 5–8, or 9–11, respectively. 

### 2.3. Data Collection

The convenience and snowball sampling methods were used for data collection. Different social media platforms, including Facebook and WhatsApp, were utilized to administer this survey. The participants were requested to share the questionnaire with their friends, colleagues, and students. The participants were able to answer all of the questions by simply clicking on the link. The objectives of the study were mentioned on the first page. Moreover, information about confidentiality, the right to withdraw, consent, and voluntary participation were also provided. Only participants more than 15 years old and currently living in Pakistan were included in this study. No incentives were given to the participants. 

### 2.4. Data Analysis

Descriptive statistics were used to assess the sample characteristics (frequency and percentages). The normality of the data was determined by Kolmogorov–Smirnov and Shapiro–Wilk tests. Median and interquartile ranges (IQR) were measured as the data showed skewed distribution. For the continuous data, Mann–Whitney and Kruskal–Wallis tests were employed. The median knowledge score was calculated which was later compared with the demographics. A chi-square test was also used where applicable. The predictors of poor practices were determined using regression analysis. SPSS (SPSS Inc, version 19, IBM, Chicago, IL, USA) was used to conduct all analyses. A *p* < 0.05 was considered statistically significant. 

### 2.5. Ethical Approval

Ethical approval to conduct this study was obtained from Xi’an Jiaotong University (Ref: Phar-2020-012).

## 3. Results

### 3.1. Demographic Information

A total of 1257 participants completed the online questionnaire. Among them, 700 (55.7%) were female, 910 (72.1%) were single, and 544 (43.3%) held a bachelor’s degree. Most of the participants, i.e., 928 (73.8) of them, were between 16 to 29 years old, and 691 (55%) were students. More than half or 771 (61.3%) of the survey participants were from the Punjab province. Detailed demographic information is provided in Table 2.

### 3.2. Knowledge of the Participants

**Figure 1:**
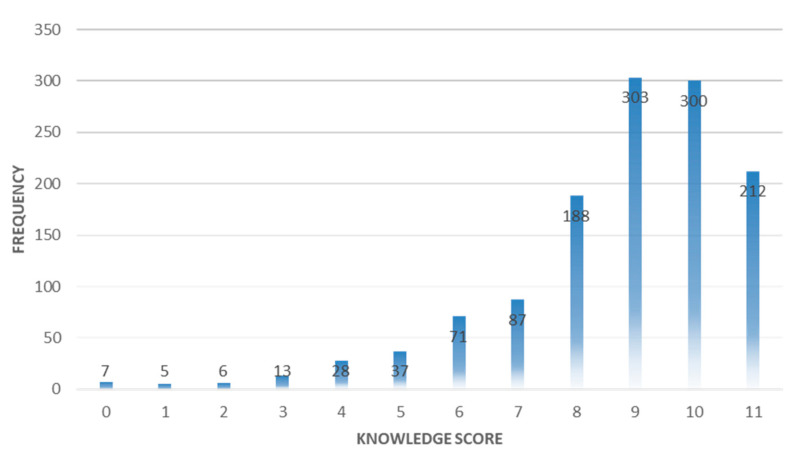
Knowledge score of the participants towards COVID-19.

Most of the participants had good (64.8%) or average (30.5%) COVID-19-related knowledge (Figure 1). A large number of participants (89.2%) were able to recognize the symptoms of COVID-19. They knew that there was no cure for COVID-19 at the moment; however, they were aware that most patients could recover by early diagnosis coupled with supportive therapy. The participants (81.3%) believed that wearing a mask could help in the prevention of COVID-19. Moreover, the majority of the participants (89.6%) were of the view that patients infected with COVID-19 should be isolated immediately (Table 3). However, more than half of the participants (54.7%) said that the virus could not be transmitted from an infected patient who did not have any fever. The median knowledge score was found to be statistically significant with age, gender, education, marital status, occupation, and living place (Table 2). 

Male respondents were significantly more knowledgeable than females (Median = 1, IQR = 0 vs. Median = 1, IQR = 0; *p* < 0.001). The participants aged 16–29 years had the highest knowledge among the other age group (Median = 1, IQR = 0 vs. Median = 1, IQR = 0; *p* < 0.001). Besides, participants who were employed had more correct answers compared with students (Median = 1, IQR = 0 vs. Median = 1, IQR = 0; *p* < 0.001). 

### 3.3. Attitude of Participants

A large number of participants held an optimistic attitude toward COVID-19 outbreaks. In essence, 74.0% thought that COVID-19 would be successfully controlled, whereas a few participants responded that they “disagree” (15.6%) or were neutral (10.4%) toward this question. The attitude toward winning the battle against COVID-19 was noted to differ significantly in terms of education and residence (*p* < 0.05). Nearly 80% (77.0%) of the participants were confident that Pakistan would control the outbreak of COVID-19 (Table 4). This level of confidence depended on their place of living and was significantly higher in participants with higher education, such as a master’s degree (*p* < 0.05). 

### 3.4. Practices of Participants

A vast majority of participants (82.1%) had not visited any crowded area and wore a mask when they moved out of their homes (85.8%). Moreover, 88.1% of the survey participants washed their hands regularly for twenty seconds (Table 5). The rates of these three practices were found to vary significantly with demographic variables, including age, gender, education, marital status, occupation, and residence (*p* < 0.05). 

Several factors significantly influenced the practices of the participants, as revealed by the logistic regression analysis. Gender (OR 0.384, 95% CI 0.285–0.518; *p* < 0.05), age (OR 0.168, 95% CI 0.106–0.265; *p* < 0.05) marital status (OR 0.206, 95% CI 0.109–0.391; *p* < 0.05), education (OR 3.083, 95% CI 2.282–4.165; *p* < 0.05), and place of residence (OR 0.150, 95% CI 0.106–0.213; *p* < 0.05) were significantly associated with visiting a crowded area. The detailed results are presented in Table 6.

## 4. Discussion

The world is facing severe life-threatening effects due to the recent outbreak of COVID-19. To the best of our knowledge, the current study, which employed the convenience and snowball sampling techniques, is the first to report the perspective of Pakistani residents toward COVID-19. This study revealed that the COVID-19-related knowledge of most of the study participants was good, as 64.8% answered most of the questions related to the disease correctly. Moreover, their attitudes and practices toward COVID-19 were optimal. 

The high rate of correct responses of the study participants was surprising, as this study was undertaken during the initial phase of the COVID-19 outbreak. However, this may be because most of the participants in this study were well educated (bachelor’s degree = 43.3% and master’s degree = 37.5%). Additionally, due to a rapidly changing situation and overwhelming news related to COVID-19 in Pakistan and worldwide, this survey population may have engaged themselves in understanding the basics of COVID-19 from numerous informative channels, including Pakistani television (PTV) and the official website of the government of Pakistan. Moreover, awareness campaigns using print and electronic mediums have also been launched by the government to help improve the understanding of the general public about COVID-19. This is further affirmed by the significant association between knowledge score and education. A recent study conducted in China also found similar results [17].

An optimistic attitude was observed among survey participants toward COVID-19, as more than seventy percent believed that COVID-19 would be controlled successfully (74.0%), and Pakistan would be able to win the battle against this deadly virus (77.0%). This optimistic attitude of the participants may be due to the unprecedented preventive measures that the government of Pakistan took once COVID-19 reached the country. First, the government shut down its flight operation and then imposed a lockdown in most of the regions. Besides, all educational institutes, including schools, colleges, and universities, were closed [23]. All non-essential businesses were also prohibited. Moreover, travel restrictions were also applied. This has surely boosted the confidence of the survey respondents in the belief that COVID-19 would be contained. Furthermore, the participants’ knowledge about COVID-19 was higher, which also confirms this speculation. 

Most of the survey participants avoided visiting any crowded place and wore masks when they left their homes to help prevent the spread of COVID-19. This is possibly due to extensive government broadcasting about the transmission of the virus, which can easily occur via respiratory droplets from infected to healthy individuals; due to the ban on public gatherings; and because of the participants’ good knowledge. Despite this, a handful of the participants (17.9%) had visited crowded places. This risky behavior was observed among male participants aged 16–29 years and those who were unmarried. This risk-taking attitude among young people has been well demonstrated in previous studies [24,25]. A significant link was found between students and going to a crowded place, which may be explained by their young age. A few participants (14.2%) avoided wearing masks. This may be attributed to the less serious situation of the COVID-19 outbreak in their respective regions. Secondly, the masks were also not available in some areas of Pakistan due to their huge demand [26].

In this study, 88.1% of the participants practiced frequent handwashing. This practice has already been advised by the WHO to limit the spread of COVID-19 [27,28]. Moreover, several studies have also advocated that handwashing with soap effectively removes the virus [29,30]. 

The participation of the young and students was higher in this study. However, this could be explained in a way that, in Pakistan, there are 76 million (36.2%) people who have internet access, and a recent survey carried out by the Pakistan Telecommunication Authority (PTA) concluded that most of the participants (63.0%) were 20–25 years old [31]. 

This study has several limitations that should be considered. First, the sample size is not large enough to depict the views of all the people of Pakistan and, therefore, its generalizability is limited. However, this is an exploratory study that could help the government understand the knowledge, attitudes, and practices of the general public. Secondly, most of the participants in this study were young people and students. Therefore, there is a need to conduct a study for older people who are more vulnerable to contract this disease. Third, the survey was administered online by using social media platforms, which may show bias; however, due to the lockdown in most parts of the country, it was difficult to field this survey offline. Despite all the above limitations, this study is the first to highlight the knowledge and behavior of the people of Pakistan towards COVID-19.

## 5. Conclusions

This rapid online survey showed that the knowledge of residents of Pakistan was good in several aspects, and gender, marital status, education, and residence were observed to have a significant association with the median knowledge score. Moreover, the attitudes and practices of the participants toward the COVID-19 outbreak were reasonable, as most of them were involved in the regular practices of avoiding crowded areas and hand washing. Likewise, a large number of participants believed that Pakistan would be able to eradicate COVID-19 successfully. The government should plan educational sessions for less knowledgeable people to enhance their knowledge, which will subsequently improve their attitudes and practices towards COVID-19. More studies with nationwide sampling are warranted to validate the current findings. 

## Figures and Tables

**Table 1:** Statistics of coronavirus (COVID-19) in Pakistan (as of 22 April 2020) [11].

Province	Total Cases	Active Cases	Total Deaths	Total Recoveries
Azad Jammu and Kashmir	51	28	0	23
Balochistan	495	322	6	167
Gilgit-Baltistan	290	89	3	198
Islamabad	194	165	3	26
Khyber Pakhtunkhwa	1345	930	80	335
Punjab	4328	3535	51	742
Sindh	3373	2639	69	665
Total	10,076	7708	212	2156

**Table 2:** Demographic characteristics of the participants and the knowledge score (*n* = 1257).

Variable	Frequency (n)	Percentage (%)	Median (IQR)	*p*-Value
**Gender**				
Male	557	44.3	1.0 (0.00)	<0.001
Female	700	55.7	1.0 (0.00)	
**Age (years)**				
16–29	928	73.8	1.0 (0.00)	<0.001
≥30	329	26.2	1.0 (0.00)	
**Marital status**				
Single	910	72.4	1.0 (0.00)	<0.001
Married	300	23.9	1.0 (0.00)	
Others ^	47	3.7	1.0 (1.00)	
**Education**				
Matriculation or below	87	6.9	1.0 (0.00)	
Higher secondary school	154	12.3	1.0 (0.00)	
Bachelor degree	544	43.3	1.0 (0.00)	
Master degree	472	37.5	1.0 (0.00)	<0.001
**Occupation**				
Employed	386	30.7	1.0 (0.00)	0.011
Unemployed	180	14.3	1.0 (0.00)	
Student	691	55.0	1.0 (0.00)	
**Residence**				
Punjab	771	61.3	1.0 (0.00)	0.033
Sindh	86	6.9	1.0 (0.00)	
KPK	107	8.5	1.0 (0.00)	
Others ^^	293	23.3	1.0 (0.00)	

^ Others include widow, separated, and divorced; ^^ Others include Balochistan, and three territories, including Islamabad Capital Territory, Gilgit–Baltistan, Azad Jammu, and Kashmir.

**Table 3:** Knowledge of the participants towards COVID-19 (*n* (%)).

Question	Yes	No	Unclear	Correct Rate
The main clinical symptoms of COVID-19 are fever, fatigue, and dry cough.	1121 (89.2)	104 (8.3)	32 (2.5)	1121 (89.2)
Unlike the common cold, stuffy nose, runny nose, and sneezing are less common in persons infected with the COVID-19 virus.	841 (66.9)	271 (21.6)	145 (11.5)	841 (66.9)
There currently is no effective cure for COVID-19, but early symptomatic and supportive treatment can help most patients recover from the infection.	1126 (89.6)	74 (5.9)	57 (4.5)	1126 (89.6)
Not all persons with COVID-19 will develop severe cases. Only those who are elderly, have chronic illnesses, and are obese are more likely to be severe cases.	998 (79.4)	166 (13.2)	93 (7.4)	998 (79.4)
Persons with COVID-19 cannot infect the virus to others when a fever is not present.	380 (30.2)	687 (54.7)	190 (15.1)	687 (54.7)
The COVID-19 virus spreads via respiratory droplets of infected individuals.	1038 (82.6)	219 (17.4)	117 (9.3)	1038 (82.6)
Ordinary residents can wear general medical masks to prevent the infection by the COVID-19 virus.	1022 (81.3)	172 (13.7)	63 (5.0)	1022 (81.3)
It is not necessary for children and young adults to take measures to prevent the infection by the COVID-19 virus.	427 (34.0)	749 (59.6)	81 (6.4)	749 (59.6)
To prevent the infection by COVID-19, individuals should avoid going to crowded places such as train stations and avoid taking public transportations.	1118 (88.9)	85 (6.8)	54 (4.3)	1118 (88.9)
Isolation and treatment of people who are infected with the COVID-19 virus are effective ways to reduce the spread of the virus.	1125 (89.5)	66 (5.3)	66 (5.3)	1125 (89.5)
People who have contact with someone infected with the COVID-19 virus should be immediately isolated in a proper place. In general, the observation period is 14 days.	1126 (89.6)	64 (5.1)	67 (5.3)	1126 (89.6)

**Table 4:** Attitude of the participants about COVID-19 by demographics.

Variables	Attitude Frequency (%)				
	Controlling COVID-19 Successfully			Winning Confidence	
Variables	Agree	Disagree	Do not Know	Yes	No/Maybe
**Gender**					
Male	418 (75.0)	74 (13.3)	65 (11.7)	438 (78.6)	119 (21.4)
Female	512 (73.1)	122 (17.4)	66(9.4)	530 (75.7)	170 (24.3)
**Age (years)**					
16–29	685 (73.8)	142 (15.3)	101 (10.9)	727 (78.3)	201 (21.7)
≥30	245 (74.5)	54 (16.4)	30 (9.1)	241 (73.3)	88 (26.7)
**Marital status**					
Married	217 (72.3)	50 (16.7)	33 (11.0)	224 (74.7)	76 (25.3)
Single	677 (74.4)	137 (15.1)	96 (10.5)	710 (78.0)	200 (22.0)
Others ^	36 (76.6)	9 (19.1)	2 (4.3)	34 (72.3)	13 (27.7)
**Education**					
Matriculation or below	66 (75.9)	15 (17.2)	6 (6.9) ***	63 (72.4)	24 (27.6) **
Higher secondary school	89 (57.8)	38 (24.7)	27 (17.5)	100 (65.0)	54 (35.0)
Bachelor degree	397 (73.0)	85 (15.7)	62 (11.3)	425 (78.1)	119 (21.9)
Master degree	378 (80.1)	58 (12.3)	36 (7.6)	380 (80.5)	92 (19.5)
**Occupation**					
Employed	294 (76.2)	57 (14.8)	35 (6.4)	301 (78.0)	85 (22.0)
Unemployed	126 (70.0)	27 (15.0)	27 (15.0)	131 (72.8)	49 (27.2)
Student	510 (73.8)	112 (16.2)	69 (10.0)	536 (77.6)	155 (22.4)
**Residence**					
Punjab	545 (70.8)	140 (18.1)	86 (11.1) *	600 (77.8)	171 (22.8)
Sindh	65 (76.6)	11 (12.8)	10 (11.6)	65 (75.6)	21 (24.4)
KPK	87 (81.3)	11 (10.3)	9 (8.4)	86 (80.4)	21 (19.6)
Others ^^	233 (79.5)	34 (11.6)	26 (8.9)	217 (76.1)	76 (25.9)

^ Others include widow, separated, and divorced; ^^ Others include Balochistan, and three territories, including Islamabad Capital Territory, Gilgit–Baltistan, Azad Jammu, and Kashmir. * *p* < 0.05; ** *p* < 0.01; *** *p* < 0.001.

**Table 5:** Practices of the participants during the COVID-19 outbreak, by demographics.

Variables	Practices n (%)					
	Visit Crowdy Place		Wearing a Mask		Hand Washing	
	Yes	No	Yes	No	Yes	No
**Gender**						
Male	143 (25.7)	414 (74.2) ***	488 (87.6)	69 (12.4)	475 (85.3)	82 (14.7) **
Female	82 (11.7)	618 (88.3)	591 (84.4)	109 (15.6)	632 (90.3)	68 (9.7)
**Age (years)**						
16–29	121 (13.0)	807 (87.0) ***	800 (86.2)	128 (13.8)	816 (87.9)	112 (12.1)
30–49	104 (31.6)	225 (68.4)	279 (84.8)	50 (15.2)	291 (88.5)	38 (11.5)
**Marital status**						
Married	61 (20.3)	239 (79.7) ***	261 (87.0)	39 (13.0)	271 (90.3)	29 (9.7)
Single	138 (15.2)	772 (84.8)	780 (85.7)	130 (14.3)	796 (87.5)	114 (12.5)
Others ^	26 (55.3)	21 (44.7)	38 (80.9)	9 (19.1)	40 (85.1)	7 (14.9)
**Education**						
Matriculation or below	42 (59.8)	45 (51.7) ***	75 (86.2)	12 (13.8) ***	78 (89.7)	9 (10.3) ***
Higher secondary school	42 (27.3)	112 (72.7)	112 (72.7)	42 (27.3)	114 (74.0)	40 (26.0)
Bachelor degree	80 (14.7)	464 (85.3)	480 (88.2)	64 (11.8)	486 (89.3)	58 (10.7)
Master degree	61 (12.9)	411 (87.1)	412 (87.3)	60 (12.7)	429 (90.9)	43 (9.1)
**Occupation**						
Employed	91 (23.6)	295 (76.4) **	331 (85.8)	55 (14.2)	345 (89.4)	41 (10.6)
Unemployed	30 (16.7)	150 (83.3)	158 (87.8)	22 (12.2)	163 (90.6)	17 (9.4)
Student	104 (15.1)	587 (84.9)	590 (85.4)	101 (14.6)	599 (86.7)	92 (13.3)
**Residence**						
Punjab	63 (8.2)	708 (91.8) ***	647 (83.9)	124 (16.1) *	668 (86.6)	103 (13.4)
Sindh	26 (30.2)	60 (69.8)	73 (84.9)	13 (15.1)	76 (88.4)	10 (11.6)
KPK	27 (25.2)	80 (74.8)	94 (87.9)	13 (12.1)	97 (90.7)	10 (9.3)
Others ^^	109 (37.2)	184 (62.8)	265 (90.4)	28 (9.6)	266 (90.8)	27 (9.2)

^ Others include widow, separated, and divorced; ^^ Others include Balochistan, and three territories, including Islamabad Capital Territory, Gilgit–Baltistan, Azad Jammu, and Kashmir. * *p* < 0.05; ** *p* < 0.01; *** *p* < 0.001.

**Table 6:** Predictors of poor practices (going to a crowded place) towards COVID-19.

Variable	OR (95% CI)	*p*-Value
**Going to a crowded place**		
Gender (Male vs. female)	0.384 (0.285–0.518)	<0.001
Age group (16–29 vs. ≥30 years)	3.083 (2.282–4.165)	<0.001
Marital status (single vs. others **^**)	0.206 (0.109–0.391)	<0.001
Marital status (married vs. others **^**)	0.144 (0.079–0.264)	<0.001
Education (matriculation or below vs. master degree)	6.289 (3.817–10.359)	<0.001
Education (higher secondary school vs. master degree)	2.527 (1.619–3.943)	<0.001
Occupation (employed vs. students)	1.741 (1.272–2.384)	0.001
Residence (Punjab vs. others ^)	0.150 (0.106–0.213)	<0.001
Residence (KPK vs. others ^)	0.570 (0.347–0.936)	0.026

**^** Others include widow, separated, and divorced; **^^** Others include Balochistan, and three territories, including Islamabad Capital Territory, Gilgit–Baltistan, Azad Jammu, and Kashmir.
